# Expression of Leptin Receptor and Effects of Leptin on Papillary Thyroid Carcinoma Cells

**DOI:** 10.1155/2019/5031696

**Published:** 2019-02-14

**Authors:** Marilena Celano, Valentina Maggisano, Saverio Massimo Lepore, Marialuisa Sponziello, Valeria Pecce, Antonella Verrienti, Cosimo Durante, Marianna Maranghi, Piernatale Lucia, Stefania Bulotta, Giuseppe Damante, Diego Russo

**Affiliations:** ^1^Department of Health Sciences, University “Magna Graecia” of Catanzaro, 88100 Catanzaro, Italy; ^2^Department of Translational and Precision Medicine, “Sapienza” University of Rome, 00161 Rome, Italy; ^3^Department of Medical Area, University of Udine, 33100 Udine, Italy

## Abstract

**Background:**

Obesity has been hypothesized to contribute to the aggressiveness of thyroid cancer through the production of abnormal levels of serum adipokines. Leptin receptor (OB-R) expression has also been documented in papillary thyroid cancer (PTC).

**Aim:**

In this translational study, we analyzed *in vitro* the effects of leptin on the growth and migration of thyroid cancer cells (TPC-1 and K1), the molecular mechanisms underlying leptin's action, and the influence of prolonged leptin exposure on cell response to a protein kinase inhibitor lenvatinib. The expression levels of *OB-R* mRNA and protein were also investigated *in vivo* in a series of aggressive PTCs divided into two groups based on the presence of the *BRAF* mutation.

**Results:**

In TPC-1 and K1 cells, prolonged treatment with leptin (500 ng/ml for 96 h) resulted in a mild increase in the proliferation (about 20% over control only in K1 cells, *p* < 0.05) and in the migration of both cancer cell lines. Immunoblot analysis revealed a slight increase in the phosphorylation of AKT, but no effect on *β*-catenin and phospho-ERK expressions. The inhibitory effects of lenvatinib on the viability of both cell lines were not influenced by the leptin treatment*. OB-R* transcript (in fresh tissues) and proteins (in formalin-fixed and paraffin-embedded specimens) were expressed in all PTC tissues examined, with no significant differences between *BRAF-*mutated and *BRAF*-wild-type tumors.

**Conclusions:**

These results demonstrate leptin's role in mildly increasing the aggressive phenotype of PTC cells but without influencing the action of lenvatinib. Further studies will clarify whether it is possible to target OB-R, expressed in all aggressive PTCs, as an adjuvant treatment approach for these malignancies.

## 1. Introduction

While the majority of differentiated thyroid cancers are responsive to the current surgery/radioiodine treatments, there are subgroups of tumors which show a more aggressive behavior. In these tumors, which have lost the capacity to concentrate radioiodine, the identification of the pathogenic mechanism involved in their development and progression is fundamental in order to individualize molecular targets for novel therapeutic approaches [1–3]. The strong association between obesity and thyroid cancer and the well-known oncogenic action of some adipokines and their respective signaling pathways have suggested that the abnormal levels of adipokines associated with obesity may be a risk factor for these aggressive thyroid cancers [4]. Among adipokines, leptin, a pleiotropic molecule encoded by the obese gene with a pivotal role in regulating food intake, energy metabolism and body weight, is also thought to influence the development of many other human malignancies [5–7]. Indeed, many studies have demonstrated leptin's influence on the expression of cell cycle modulators, cancer cell proliferation and transformation, and cell migration and invasion [5–7]. In addition, an overexpression of leptin and its receptor (*OB-R*) has been reported in many cancer types, including thyroid malignancies [5–9]. In this study, we analyzed the effects of leptin on two human papillary thyroid cancer (PTC) cell lines, focusing on the molecular mechanisms underlying leptin's effects on cell viability and migration. Moreover, we checked the influence of prolonged leptin treatment on cell response to the protein kinase inhibitor (PKI) lenvatinib. The expression of the *OB-R* transcript and protein was also investigated in a series of aggressive PTCs classified as intermediate/high risk according to the 2015 ATA criteria [10].

## 2. Materials and Methods

The study design included *in vitro* and *ex vivo* experiments as described below.

### 2.1. *In Vitro* Experiments

#### 2.1.1. Thyroid Cancer Cell Lines

For *in vitro* experiments, two human PTC cell lines, K1 and TPC-1, were used. These cell lines contained the *BRAF* V600E and *RET/PTC1* mutation, respectively [11]. Cells were grown in a DMEM medium (Thermo Fisher Scientific Inc., Waltham, MA, USA), supplemented with a 10% foetal bovine serum (FBS) (Thermo Fisher Scientific), penicillin (100 IU/ml), streptomycin (100 mg/ml), and amphotericin B (2.5 mg/ml) (Sigma-Aldrich, Milan, Italy), and maintained at 37°C in a humidified atmosphere containing 5% CO_2_. Short tandem repeat profiling was used to authenticate these cell lines. Cultured cells were treated with 200 or 500 ng/ml of leptin (Sigma-Aldrich) for 96 h (Leptin), 50 *μ*M of lenvatinib (Selleckchem, Aurogene Srl, Rome, Italy) (Lenvatinib) for 24 h, or with 500 ng/ml of leptin for 96 h plus 50 *μ*M of lenvatinib for an additional 24 h (Leptin+Lenvatinib). Untreated cells were used as a control (ctrl).

#### 2.1.2. Cell Viability and Migration Assay

Cell viability was analyzed by an MTT assay [12]. Briefly, cells were seeded in 96-well plates at a density of 3.0 × 10^3^ in a medium with 10% FBS. After 24 h, the growth medium was replaced by a fresh medium containing 0.1% FBS and supplemented with 200 or 500 ng/ml leptin (Sigma-Aldrich) for 96 h with or without 50 *μ*M of the PKI lenvatinib (Selleckchem, Aurogene) for an additional 24 h. The solubilized product was then quantified with a microplate spectrophotometer (xMark, Bio-Rad, Milan, Italy) at a wavelength of 540 nm and a reference wavelength of 690 nm. Results are expressed as percentages over untreated cells. For the migration assay, after the treatment with 200 or 500 ng/ml of leptin for 96 h, 60 × 10^3^ cells suspended in a serum-free medium containing 1% BSA were plated in the upper compartments of the Boyden chamber (8 *μ*m membranes) (Costar, EuroClone, Milan, Italy). In the bottom wells, 600 *μ*l of the medium containing 10% FBS was added as a chemotactic agent. After 6 h, migrated cells were fixed and stained with a Diff-Quick stain (Biomap snc, Monza, Italy). Migration was quantified by viewing eight separate fields using a microscope (objective 10x) with an eyepiece equipped with a counting grid. Results are expressed as percentages over control.

#### 2.1.3. Immunoblot Analysis

Total proteins were extracted from K1 and TPC-1 cell lysates as previously described [13]. 20 *μ*g of proteins was run on a 9% SDS-PAGE gel, transferred to the PVDF membranes (VWR, Milan, Italy), blocked with PBS-Triton/milk (PBS 1X, 0.1% Triton, and 5% nonfat dry milk) for 1 h at room temperature, and incubated overnight with the following antibodies: anti-*β*-catenin (1 : 1000) (Cell Signaling, Danvers, MA, USA), anti-AKT and anti-ERK (1 : 1000) and anti-phospho-AKT and anti-phospho-ERK (1 : 500) (Cell Signaling and Santa Cruz, Heidelberg, Germany, respectively), anti-OB-R (1 : 500) (Abcam, Cambridge, United Kingdom), and anti-GAPDH (1 : 10000) (Thermo Fisher Scientific). The membranes were washed in PBS-Triton and incubated with a horseradish peroxidase-conjugated anti-rabbit or anti-mouse antibody (Transduction Laboratories, Lexington, KY, USA) in PBS-Triton/milk, diluted to 1 : 5000, 1 : 10000, 1 : 20000, 1 : 30000, or 1 : 8000. The proteins were visualized by chemiluminescence using the Western blot detection system ECL Plus (PerkinElmer, Monza, Italy).

### 2.2. Ex Vivo Experiments

#### 2.2.1. Patients and Tissue Specimens

Twenty-three Italian patients who underwent surgery for PTC from July 2010 to December 2013 were enrolled in this retrospective study. The inclusion criteria were as follows: (a) histological diagnosis of papillary thyroid cancer, (b) intermediate or high risk for recurrence according to the “2015 ATA Guidelines for the Management of Adult Patients with Thyroid Nodules and Differentiated Thyroid Cancer” [10], and (c) percentage of tumor cells in tissues used for molecular analysis higher than 60% after histological review.

After thyroidectomy, samples of thyroid tumor tissues were collected and immediately frozen. In all samples, *BRAF* mutational status was determined by the Sanger sequencing, as previously described [14]. Clinicobiological features including sex, age, tumor size and foci, extrathyroidal extension, lymph node metastases, patient outcome, body mass index (BMI), and *BRAF* mutational status have been summarized in Table 1. Fresh-frozen tumor tissues from the 23 selected patients were used for gene expression analysis. Formalin-fixed and paraffin-embedded (FFPE) tumor tissues from a selection of 10 patients were analyzed by immunohistochemistry. All patients signed an informed consent form at “Sapienza” University Hospital of Rome (Italy), and the study protocol was approved by the local institutional medical ethics committee.

#### 2.2.2. Real-Time PCR Analysis

TRIzol reagent (Thermo Fisher Scientific) was used for RNA isolation from tissues. 1 *μ*g of total RNA was reverse-transcribed with a High-Capacity cDNA Reverse Transcription Kit (Thermo Fisher Scientific), and *OB-R* expression levels were quantified by real-time PCR in a 7900HT Fast Real-Time PCR System (Thermo Fisher Scientific) as previously described [15]. Each sample was analyzed in triplicate and normalized on *ACTB* mRNA content. Predesigned TaqMan Assays (probe and primer sets) for *OB-R* (Hs00900242_m1; it recognizes all the six *OB-R* isoforms: NM_001003679.3, NM_001003680.3, NM_001198687.1, NM_001198688.1, NM_001198689.1, and NM_002303.5) and *ACTB* (Hs99999903_m1) were purchased from Thermo Fisher Scientific. Data analyses were carried out using SDS 2.4 software (Thermo Fisher Scientific), and results were determined by the comparative 2^−ΔCt^ method and shown as relative expression normalized to a calibrator sample group.

#### 2.2.3. Immunohistochemical Analysis

Paraffin-embedded sections (5 *μ*m thick) from 10 patients were dewaxed, rehydrated, and treated with a citrate buffer (0.01 M, pH 6) to retrieve epitope. H_2_O_2_ was then used to block endogenous peroxidase activity. Immunodetection was carried out using an anti-OB-R antibody (1 : 200 dilution) (Cell Signaling, Danvers), and, after washing, incubation with a biotinylated goat anti-polyvalent antibody (Detection IHC kit, Abcam) was performed. Finally, staining was visualized using 3,3-diaminobenzidine tetrahydrochloride (Detection IHC kit, Abcam). The sections were slightly counterstained with Mayer hematoxylin (Carlo Erba Reagents S.r.l., Milan, Italy) and were analyzed using a binocular microscope with a 20x objective, a digital image capture computer system, and the software supplied with the microscope.

### 2.3. Statistical Analysis

Data were analyzed by one-way ANOVA followed by the Tukey-Kramer multiple comparisons test. Student's *t*-test was used to evaluate the intergroup differences. All results are expressed as mean ± standard deviation (SD) and were considered statistically significant with *p* values lower than 0.05. All statistical analyses were performed using GraphPad Prism version 5.0 statistical software (GraphPad Software Inc., San Diego, CA, USA).

## 3. Results

### 3.1. Effects of Leptin on Thyroid Cancer Cells *In Vitro*

**Figure 1:**
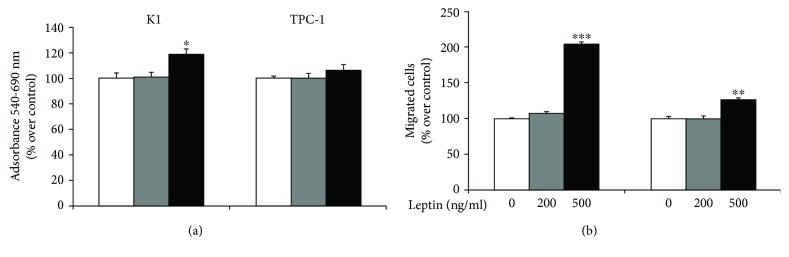
Effects of leptin on the proliferation and migration properties of K1 and TPC-1 cells. (a) Cell viability was evaluated by MTT after 96 h of incubation with 200 or 500 ng/ml leptin. Results are mean ± SD of three independent experiments performed in eigthplicate. (b) After 96 h of treatment with 200 or 500 ng/ml leptin, cells were prepared for migration assays as indicated in Materials and Methods. After 6 h, filters were stained and photographed at 10x magnification and cells counted. Differences were evaluated with Student's *t*-test. ^∗^ *p* < 0.05, ^∗∗^ *p* < 0.01, ^∗∗∗^ *p* < 0.001 vs. untreated cells.

**Figure 2:**
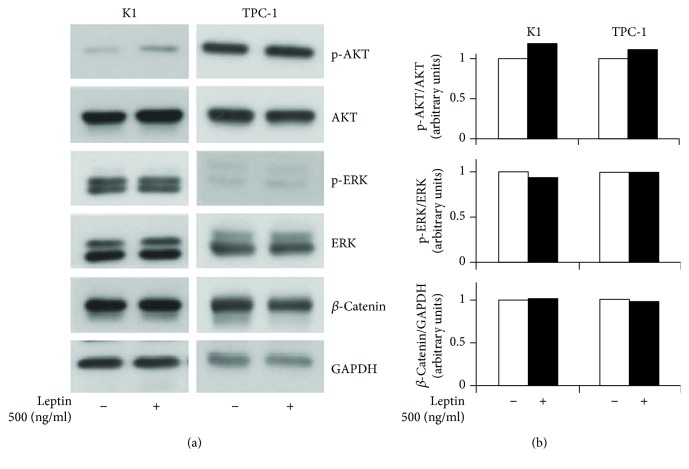
Effects of leptin treatment on signaling pathways of K1 and TPC-1 cells. (a) Immunoblot analysis of *β*-catenin, phosphorylated AKT (p-AKT) and AKT, and phosphorylated ERK (p-ERK) and ERK in K1 and TPC-1 cells after leptin treatment. GAPDH was used as a loading control. Experiments were performed as described in Materials and Methods. (b) Densitometric analysis from a representative immunoblot of p-AKT/AKT, p-ERK/ERK, and *β*-catenin. Values are expressed as a ratio over the loading control (arbitrarily assigned as 1).

**Figure 3:**
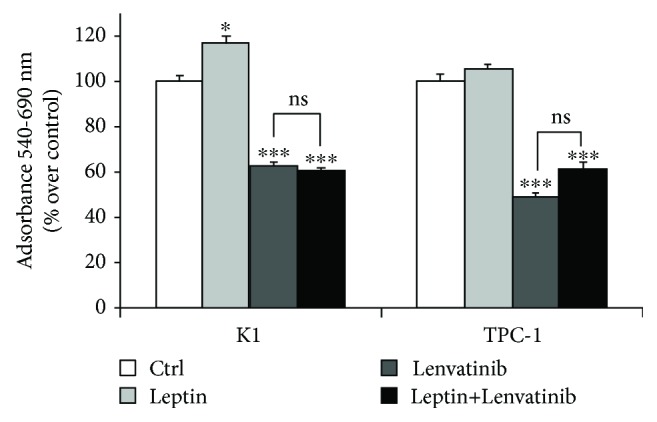
Effects of leptin on the treatment with lenvatinib on K1 and TPC-1 cells. After 96 h of treatment with 500 ng/ml leptin, selected cells were incubated or not with 50 *μ*M lenvatinib for 24 h and viability was evaluated by an MTT assay. Results are mean ± SD of three independent experiments performed in eigthplicate. Statistical analysis was performed using the one-way ANOVA test. ^∗^ *p* < 0.05, ^∗∗∗^ *p* < 0.001 vs. untreated cells (indicated as ctrl). ns: not significant.

We first analyzed the effects of leptin on K1 and TPC-1 cells. In both cell lines, similar levels of OB-R were detected by the immunoblot analysis (Supplementary Figure 1). A prolonged exposure (96 h) to leptin induced a slight but significant increase in the cell viability of K1 cells (about 20% over control, *p* < 0.05) only using 500 ng/ml of this adipokine (Figure 1(a)). In the same experimental conditions, 500 ng/ml of leptin enhanced the migration of both PTC cell lines (about 100% and 30% over control in K1 and TPC-1, *p* < 0.001 and <0.01, respectively) (Figure 1(b)). To elucidate the molecular mechanisms of leptin effects on our PTC cells, we analyzed the phosphorylation levels of ERK and AKT, together with those of *β*-catenin. Using immunoblot followed by a densitometric analysis (Figures 2(a) and 2(b)), we found that prolonged exposure to leptin promoted a slight increase in the phosphorylation of AKT but did not affect *β*-catenin and phospho-ERK expressions. We then tested the effects of prolonged leptin treatment on the action of the PKI lenvatinib. As shown in Figure 3, we observed that pretreatment with leptin did not influence the effects of lenvatinib on the viability of both K1 and TPC-1.

### 3.2. Expression of Leptin Receptor in Thyroid Cancer Tissues

**Figure 4:**
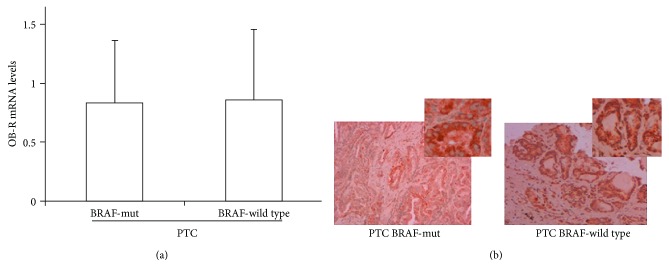
Expression of leptin receptor gene and protein in PTC tissues. (a) Expression levels of *OB-R* gene in *BRAF* V600E*-*mut and *BRAF*-wild-type PTCs. Relative expression levels are reported as mean ± SD normalized to a calibrator sample group. (b) A representative immunohistochemical analysis of OB-R expression in *BRAF-*mut and *BRAF*-wild-type PTCs.

Finally, we investigated the expression of OB-R in an Italian cohort of aggressive PTCs (Table 1). We found that *OB-R* was expressed in all tumor tissues. As shown in Figure 4(a), we did not find significant differences between the *OB-R* mRNA values detected in the subgroup of tumors carrying the *BRAF* V600E mutation, compared with those of PTCs with wild-type *BRAF*. The same observation was confirmed by the immunohistochemical analysis of the OB-R protein expression (Figure 4(b)).

## 4. Discussion

The search for novel molecular targets in aggressive DTCs unresponsive to current treatments and recent reports on the role of leptin and OB-R in some malignancies prompted us to investigate the possibility of targeting OB-Rs in thyroid cancer. Thus, in the present study, the role of prolonged leptin exposure in tumor progression and/or expansion, as it may occur *in vivo* in TC patients with high BMIs, was first analyzed in two PTC-derived cell lines carrying *RET/PTC1* (TPC-1) and *BRAF* (K1) mutations which both express OB-R. We found that prolonged treatment with high doses of leptin, as it may be observed only in obese individuals, slightly stimulated the proliferation of only K1 cells, whereas a significant increase in migration was observed in both cell lines in the same experimental conditions. The differences in the mutation drivers may explain the different behavior of our PTC cells; however, such findings demonstrate that leptin mildly increases the aggressive phenotype of these cancer cells, which is in line with some previous studies on tumor cells of thyroid [16] and breast origin [17]. The molecular mechanism underlying leptin effects was investigated by focusing on the two main oncogenic pathways activated in thyroid tumorigenesis [18, 19] through the measurement of phospho-ERK and phospho-AKT expressions. Our findings demonstrated the involvement of AKT phosphorylation, with no effects on the expression of phospho-ERK. Also, expression of *β*-catenin, whose elevated levels have recently been associated with TC recurrence [20], was not modified by treating the cells with leptin. A reduction in the AKT-mediated pathway has also been described by Uddin et al. and Cheng et al. by using different exposure times and concentrations, and, in some cases, even different thyroid cancer cell lines [9, 16]. In the latter study, ERK phosphorylation was affected after 15 min of leptin treatment, an experimental condition which differs greatly from the prolonged leptin treatment performed in our study.

Another important finding of our study is the potential influence of prolonged exposure to high levels of leptin on the action of lenvatinib, a PKI approved for the treatment of radioiodine refractory thyroid cancer [21] which has also been shown to block the proliferation of some thyroid cancer cell lines *in vitro* [22]. Interestingly, the effects of lenvatinib on the viability of both K1 and TPC-1 cell lines were not modified by pretreatment with leptin. At present, an association between high BMI or leptin serum levels and resistance or intolerance to lenvatinib has never been investigated. However, it cannot be excluded that a complete inhibition of leptin-induced effects on thyroid cancer cells may increase the effects of PKIs and thus decrease the dosage needed to be effective. As a subsequent step, various compounds able to block OB-R [17, 23] could be investigated together with PKIs as a novel therapeutic approach for radioiodine-refractory thyroid cancer.

Finally, we investigated the expression of OB-R in an Italian cohort of selected aggressive PTCs classified as intermediate or high risk according to the ATA criteria, and all expressed high levels of fibronectin [24]. We found that OB-R were expressed in all tumor tissues. However, no significant correlation was found between *OB-R* expression levels and BMI values, the presence of lymph node metastases, or patient outcome (data not shown). These results confirm those of Zhang et al., which were obtained with a larger series of patients [25]. In addition, we did not find significant differences between *OB-R* mRNA and protein levels in the subgroup of PTC tumors carrying the *BRAF* V600E mutation, proposed as a hallmark of tumor aggressiveness [26, 27], and PTCs with wild-type *BRAF*. Although Uddin et al. demonstrated that OB-R levels cannot be considered an independent prognostic marker to predict patient survival [4], the detection of OB-R in all aggressive PTCs in the present study suggests a possible contribution of high levels of leptin to the progression of thyroid malignancy and a potential use of OB-R as a therapeutic target for residual or recurrent lesions. Further studies which include PTC patients with other driver mutations which activate additional signaling pathways, as well as the investigation of the combinatory effects with other metabolic hormones, may help to shed more light on the role of this metabolic marker on PTC.

In conclusion, in our experimental conditions, prolonged exposure to high concentration of leptin seems to contribute mildly to increasing the aggressive phenotype of PTC cells but without influencing the action of lenvatinib. However, the expression of OB-R in aggressive PTCs suggests their use as a possible target in an adjuvant approach for thyroid cancer.

## Figures and Tables

**Table 1:** Clinicobiological features of PTC.

Characteristics	Study cohort (*n* = 23)
Sex: male/female	7/16
Median age at diagnosis, years (range)	45.4 (19-71)
Median tumor size^∗^, mm (range)	17.3 (7-45)
Tumor foci^∗^: unifocal/multifocal	18/4
Extrathyroidal extension: no/yes	8/15
Lymph node metastases^∗^: no/yes	9/13
Outcome: NED/BED-SED^∗∗^	9/3
Median BMI, kg/m^2^ (range)^∗∗∗^	25.96 (20.65-32.91)
Mutational status: *BRAF* mutated/wild type	17/6

^∗^Data not available for one patient. ^∗∗^Data not available for eleven patients. ^∗∗∗^Data not available for nine patients. Abbreviations: BED: biochemical evidence of disease; BMI: body mass index; NED: not evidence of disease; SED: structural evidence of disease.

## Data Availability

The data used to support the findings of this study are available from the corresponding author upon request.

## Supplementary Materials

Supplementary Materials Supplementary Figure 1: expression of leptin receptor protein in K1 and TPC-1 cells. Immunoblot and densitometric analysis of OB-R in K1 and TPC-1 cells. GAPDH was used as a loading control. Values are expressed as a ratio over the loading control (arbitrarily assigned as 1).
