# Supplementary material for: Expression of Leptin Receptor and Effects of Leptin on Papillary Thyroid Carcinoma Cells
Source: Int J Endocrinol. 2019 Feb 14;2019:5031696. doi: 10.1155/2019/5031696 (PMC6393892; doi:10.1155/2019/5031696)
Supplement: Supplementary Materials — Supplementary Figure 1: expression of leptin receptor protein in K1 and TPC-1 cells. Immunoblot and densitometric analysis of OB-R in K1 and TPC-1 cells. GAPDH was used as a loading control. Values are expressed as a ratio over the loading control (arbitrarily assigned as 1). [file 5031696.f1.pdf]

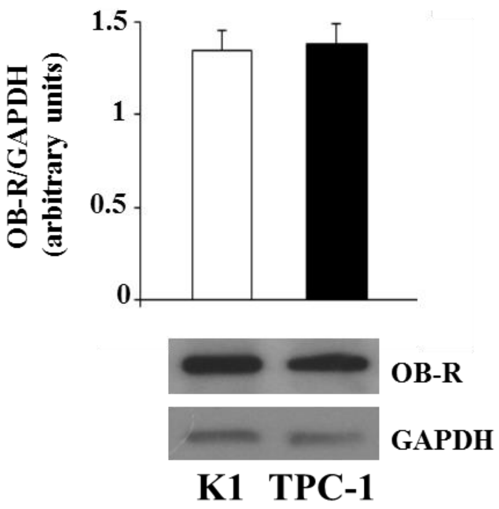

Supplementary Figure 1

Supplementary Figure 1: Expression of Leptin-receptor protein in K1 and TPC-1 cells.

Immunoblot and densitometric analysis of OB-R in K1 and TPC-1 cells. GAPDH was used as loading control. Values are expressed as ratio over the loading control (arbitrarily assigned as 1).
